# DanGer Shock‐like profile predicts the outcome in ST‐elevation myocardial infarction‐related cardiogenic shock

**DOI:** 10.1002/ehf2.15269

**Published:** 2025-03-19

**Authors:** Norman Mangner, Johannes Mierke, Dominik Baron, Felix J. Woitek, Stephan Haussig, Thomas Nowack, Ephraim B. Winzer, Julia Fischer, Robert Höllriegel, Stefanie Jellinghaus, Axel Linke

**Affiliations:** ^1^ Department for Internal Medicine and Cardiology, Heart Centre Dresden, Faculty of Medicine and University Hospital Carl Gustav Carus TUD Dresden University of Technology Dresden Germany

**Keywords:** Cardiogenic shock, ST‐elevation myocardial infarction, Mechanical circulatory support, Microaxial flow pump, DanGer Shock

## Abstract

**Aims:**

The DanGer Shock (DGS) trial demonstrated that the routine use of a microaxial flow pump (mAFP) with standard care to treat STEMI‐related cardiogenic shock (STEMI‐CS) led to a lower risk of all‐cause death at 180 days than standard care alone. We investigated the impact of patient eligibility for DGS in an all‐comers cardiogenic shock registry of patients receiving a mAFP.

**Methods and results:**

Prospective single‐centre mAFP registry including 478 CS‐patients with 225 patients having STEMI‐CS. DGS‐like was defined as STEMI‐CS, lactate ≥2.5 mmol/L, left ventricular ejection fraction < 45%, no mechanical complications, and no comatose out‐of‐hospital cardiac arrest but in‐hospital cardiac arrest with a maximum of 10 min to return of spontaneous circulation as a surrogate for medically witnessed cardiac arrest was included. The comparison group consisted of STEMI‐CS patients who did not fulfil the aforementioned criteria (DGS‐unlike). The primary outcome was 180‐day mortality. Out of 225 STEMI‐CS, 64 (28.4%) patients were considered DGS‐like. Those patients were younger, had less often received CPR before mAFP implantation, and mAFP‐support was longer. Comorbidities, baseline lactate, coronary artery disease characteristics/treatment, inotropes/vasopressors, and escalation to other mechanical circulatory support devices were not different. All‐cause mortality at 180 days was significantly lower in the DGS‐like compared to the DGS‐unlike cohort (62.5% vs. 72.0%, *P* = 0.014) as was 30‐day all‐cause mortality (48.4% vs. 70.2%, *P* < 0.001). DGS‐like remained an independent predictor of both 180‐day (HR 0.57, 95% CI 0.39, 0.83) and 30‐day mortality (HR 0.48, 95% CI 0.32, 0.72) in a multivariable analysis.

**Conclusions:**

A DGS‐like profile was associated with a lower 180‐day mortality compared with a DGS‐unlike profile in a STEMI‐CS cohort treated by mAFP.

## Graphical abstract

Flow chart illustrates the analysed patients as well as main baseline characteristics and outcomes comparing DanGer Shock‐like versus DanGer Shock‐unlike patients. The composite safety endpoint comprises severe bleeding, limb ischaemia, haemolysis, device failure, and worsening of aortic regurgitation. CS indicates cardiogenic shock.

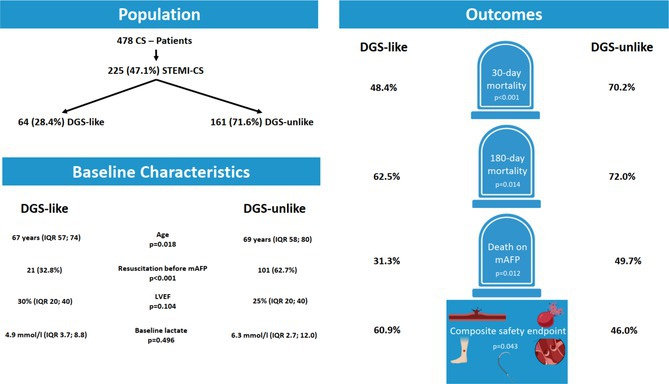

## Introduction

Cardiogenic shock (CS) is an acute haemodynamic compromise of cardiac origin characterized by low cardiac output and consequent tissue malperfusion.^1^ It can be caused by multiple diseases, present in a spectrum of severity from beginning to extremis CS according to the Society for Cardiovascular Angiography and Interventions (SCAI) SHOCK classification system, and can be divided into predominant left, right or biventricular failure.^1^, ^2^ Besides heart failure related CS, acute myocardial infarction (AMI) is a major reason for CS, and one in 10 patients with ST‐segment elevation AMI (STEMI) will develop CS.^3^, ^4^ Until recently, the only evidence‐based treatment strategy in STEMI‐CS was early revascularization of the infarct‐related artery, but it is still associated with a high mortality of 45–55%.^5^, ^6^

Continuing efforts to improve outcomes have led to a rise in the use of temporary active mechanical circulatory support (tMCS) to achieve haemodynamic stabilization in severe shock, in particular utilizing the extracorporeal membrane oxygenation (ECMO) and microaxial flow pump (mAFP). In the ECLS‐Shock trial, the routine use of ECMO did not improve clinical outcomes in patients with AMI‐CS and was associated with excess bleeding complications and limb ischaemia.^7^ Recently, the DanGer Shock (DGS) study showed that the routine use of a mAFP together with standard treatment in selected STEMI‐CS patients resulted in a lower risk of death from any cause at 180 days than standard treatment alone, with the rate of adverse events also increasing in the mAFP group.^8^ Besides important haemodynamic differences between those two devices with the mAFP unloading the left ventricle and the ECMO increasing afterload, patient selection, in particular the exclusion of comatose out‐of‐hospital cardiac arrest (OHCA), appears to be a major point contributing to the observed benefit in DGS. In a collaborative meta‐analysis of randomized controlled trials (RCTs) with individual patient data, a significant survival benefit of tMCS over the control group was observed in the selected group of patients with STEMI‐CS without high risk of neurological damage, for example, return of spontaneous circulation (ROSC) within 10 min after cardiac arrest, which corresponds to the population enrolled in DGS.^9^

The applicability and impact on mortality of such a patient selection outside of RCTs is not known but may provide an opportunity to differentiate between patients that might derive a benefit from a mAFP and those with a futile prognosis even under mAFP support. We therefore investigated the proportion of patients who would have been eligible for DGS and its impact on survival and complications in a single‐centre all‐comers registry of patients with cardiogenic shock who received a mAFP.

## Methods

### Study population.

Patient data were collected from the Dresden Impella Registry, an ongoing registry of a high‐volume centre that consecutively includes CS patients who received a mAFP. Since our centre participated in both the DGS trial^8^ and the ECLS Shock trial,^7^ patients randomized in those trials were excluded from this analysis. CS was defined by the presence of cardiac disease, systolic blood pressure less than 90 mmHg for more than 15 min or a requirement of catecholamines to maintain systolic blood pressure above 90 mmHg, and clinical signs of impaired end‐organ perfusion, as indicated by increased serum lactate levels.^10^, ^11^ Implantation of the mAFP was typically performed in a cardiac catheterization laboratory by an experienced interventional cardiologist (>20 implantations per annum). Afterwards, patients were treated on a specialized cardiac intensive care unit. This study was approved by the Ethics Committee at TU Dresden (EK 457‐122‐014).

### Definition of DanGer Shock‐like patients

DGS‐like was defined as STEMI‐CS, lactate ≥2.5 mmol/L, left ventricular ejection fraction (LVEF) < 45%, and no mechanical complications, no comatose OHCA but in‐hospital cardiac (IHCA) arrest with a maximum of 10 min to ROSC as a surrogate for medically witnessed CA was included. All other STEMI‐CS patients who did not fulfil these criteria were classified as DGS‐unlike and formed the comparison group.

### Study outcomes and variables

The primary outcome measure was all‐cause mortality at 180 days. Secondary outcomes included all‐cause mortality at 30 days, cardiopulmonary resuscitation (CPR) after mAFP, death on mAFP, successful weaning from mAFP, and length of ICU stay. Adverse events were evaluated as a composite of severe bleeding, limb ischaemia, haemolysis, device failure, and worsening of aortic regurgitation (as in the DGS trial).^8^ Moreover, the single components of the composite safety outcome, the rate and cause of stroke, the requirement for renal replacement therapy, and the occurrence of blood culture‐positive sepsis^12^ are reported. Bleeding events were adjudicated according to Global Use of Strategies to Open Occluded Arteries (GUSTO) criteria to align with the definition used in the primary report of the DGS trial.^8^ Causes of bleeding were assessed as access site‐ and non‐access site‐related. Outcome parameters were collected in‐hospital and during clinically indicated outpatient visits.

### Statistical analysis

The statistical analysis was performed using SPSS Statistics version 29.0 (IBM Corporation, Armonk, USA). Categorical variables are expressed as counts and percentages and groups were compared with the use of the chi‐squared test or Fisher exact test if the expected frequency was less than six. Continuous variables are expressed as the median with corresponding 25th and 75th percentile and groups were compared using the Mann–Whitney *U* test due to a non‐normal distribution assessed by the Shapiro–Wilk test.

Thirty‐day and 180‐day mortality were analysed according to the method of Kaplan–Meier and group comparisons were made by log‐rank test. To account for baseline differences and to receive age‐ and sex‐adjusted hazard ratios (HR) with corresponding 95% confidence interval (CI) for 30‐day and 180‐day all‐cause mortality, a stepwise backward Cox regression was performed including the factors age, sex, atrial fibrillation, CPR before mAFP, baseline lactate (≥2.5 mmol vs.<2.5 mmol/L), and DGS‐like vs. DGS‐unlike. Additionally, another Cox regression model was built including clinically relevant baseline characteristics and complications having a *P* value < 0.1 in univariable analysis to elucidate the impact of complications during treatment on 180‐day mortality. In both scenarios, a final model was calculated by including the significant variables selected by backward selection to obtain final coefficients. The Cox regression analysis for 30‐day and 180‐day mortality was chosen due to the fact that mortality is based on rates over time.

Moreover, odds ratios (OR) with corresponding 95% CI were calculated for the outcome CPR after mAFP, death on device, and successful weaning of mAFP adjusting for the before mentioned variables in a stepwise backward binary logistic regression. A final model was calculated by including the significant variables selected by backward selection to obtain final coefficients. This method was chosen due to the binary nature of these outcomes, without recording the specific time of occurrence. Finally, for length at ICU, a continuous variable, a linear regression was performed adjusting for the before mentioned variables to receive a regression coefficient beta with corresponding 95% CI. A final model was calculated by including the significant variables selected by backward selection to obtain final coefficients.

The factors included in the models were tested for collinearity, which was assumed if R was greater than 0.70 in the bivariate correlation test, the tolerance value was below 0.10, and/or the variable inflation factor (VIF) was greater than 10. No correction was performed for multiple testing and missing values were not imputed. A two‐sided *P* value < 0.05 was considered significant.

## Results

### Patient selection and baseline characteristics

Among 478 patients receiving a mAFP for CS included in the Dresden Impella Registry from January 2014 to January 2022, 225 (47.1%) patients were treated for STEMI‐CS. Among them, 64 (28.4%) and 161 (71.6%) patients were considered DGS‐like and DGS‐unlike, respectively.

Baseline and procedural characteristics are displayed in *Table* *1*. DGS‐like patients, compared to DGS‐unlike patients, were significantly younger, had less often received CPR before mAFP insertion with a shorter median time to ROSC, and received a longer mAFP support (all *P* < 0.05; *Table* *1*). All patients were treated with an Impella CP, with the exception of four patients in DGS‐unlike, who received an Impella 2.5 (*P* = 0.580; *Table* *1*). Other parameters, including comorbidities, infarct localization, LVEF, and baseline blood pressure and heart rate as well as baseline lactate, were not different between groups. However, the proportion of patients with a baseline lactate ≥2.5 mmol/L was 100% in DGS‐like patients, whereas only 75.8% of DGS‐unlike patients met this criterion (*P* < 0.001). The need for mechanical ventilation, vasopressors, and inotropes and the escalation of additional tMCS were not significantly different between DGS‐like and DGS‐unlike patients (*Table* *1*).

**Table 1:** Baseline characteristics.

	All *n* = 225	DGS‐like STEMI‐CS *n* = 64	DGS‐unlike STEMI‐CS *n* = 161	*P* value
Age, years	69 (58, 82)	67 (57, 74)	69 (58, 80)	0.018
Female sex, *n* (%)	55/255 (24.4)	15/64 (23.4)	40/161 (24.8)	0.825
Body mass index, kg/m^2^	26.7 (24.6, 29.4)	26.7 (24.2, 29.9)	26.6 (24.7, 29.0)	0.993
Arterial hypertension, *n* (%)	149/221 (67.4)	45/63 (71.4)	104/158 (65.8)	0.422
Atrial fibrillation, *n* (%)	49/225 (21.8)	19/64 (29.7)	30/161 (18.6)	0.070
Diabetes mellitus, *n* (%)	69/224 (30.8)	17/64 (26.6)	52/160 (32.5)	0.385
CKD stage ≥3, *n* (%)	58/223 (26.0)	16/64 (25.0)	42/159 (26.4)	0.828
PAD, *n* (%)	18/224 (8.0)	4/64 (6.3)	14/160 (8.8)	0.534
COPD, *n* (%)	10/224 (4.5)	2/64 (3.1)	8/160 (5.0)	0.728
Dyslipidaemia, *n* (%)	125/220 (56.8)	41/64 (64.1)	84/156 (53.8)	0.165
Previous AMI, *n* (%)	39/225 (17.3)	8/64 (12.5)	31/161 (19.3)	0.227
Previous CABG, *n* (%)	8/225 (3.6)	3/64 (4.7)	5/161 (3.1)	0.691
Previous PCI, *n* (%)	45/225 (20.0)	8/64 (12.5)	37/161 (23.0)	0.076
Previous stroke, *n* (%)	22/225 (9.8)	7/64 (10.9)	15/161 (9.3)	0.712
Mean blood pressure, mmHg	74 (60, 89)	74 (66, 84)	73 (60, 90)	0.642
Heart rate, min^−1^	95 (75, 115)	100 (80, 116)	92 (74, 110)	0.122
Arterial lactate, mmol/L	5.6 (3.2, 11.6)	4.9 (3.7, 8.8)	6.3 (2.7, 12.0)	0.496
Arterial lactate ≥2.5 mmol/L, *n* (%)	180/217 (82.9)	64/64 (100)	116/153 (75.8)	<0.001
LVEF, %	30 (20, 40)	30 (20, 40)	25 (20, 40)	0.104
Resuscitation before mAFP, *n* (%)	122/225 (54.2)	21/64 (32.8)	101/161 (62.7)	<0.001
Median time of CPR, min	20 (10, 53)	7 (5,10)	30 (15, 60)	<0.001
Coronary artery disease, *n* (%)				0.866
None	1/223 (0.4)	0/64 (0)	1/159 (0.6)	
1‐vessel	49/223 (22.0)	13/64 (20.3)	36/159 (22.6)	
2‐vessel	65/223 (29.1)	18/64 (28.1)	47/159 (29.6)	
3‐vessel	108/223 (48.4)	33/64 (51.6)	75/159 (47.2)	
Infarct‐related artery LM or LAD, *n* (%)	137/219 (62.6)	41/64 (65.1)	96/156 (61.5)	0.624
PCI treatment, *n* (%)	217/225 (96.4)	64/64 (100)	153/161 (95.0)	0.109
Median time from symptoms to mAFP, hours	2 (1.0, 3.8)	2 (1.0, 4.4)	2 (1.0, 3.5)	0.864
Duration of mAFP support, hours	34.5 (8.0, 72.9)	46.2 (19.9, 96.9)	26.7 (4.3, 69.3)	0.019
Type of mAFP, *n* (%)				0.580
Impella CP	221/225 (98.2)	64/64 (100)	0/64 (0)	
Impella 2.5	4/225 (1.8)	0/64 (0)	4/161 (2.5)	
Additional tMCS, *n* (%)				0.571
Impella RP	4/225 (1.8)	0/64 (0)	4/161 (2.5)	
V‐A ECMO	10/225 (4.4)	4/64 (6.3)	6/161 (3.7)	
Ventilation, *n* (%)				0.084
None	22/225 (9.8)	10/64 (15.6)	12/161 (7.5)	
Non‐invasive	14/225 (6.2)	4/64 (6.3)	10/161 (6.2)	
Invasive	146/225 (64.9)	34/64 (53.1)	112/161 (69.6)	
Both	43/225 (19.1)	16/64 (25.0)	27/161 (16.8)	
Any norepinephrine, *n* (%)	193/223 (86.5)	51/63 (81.0)	142/160 (88.8)	0.124
Any vasopressin, *n* (%)	81/222 (36.5)	19/63 (30.2)	62/159 (39.0)	0.218
Any dobutamine, *n* (%)	118/222 (53.2)	28/63 (44.4)	90/159 (56.6)	0.102
Any enoximone, *n* (%)	31/222 (14.0)	11/63 (17.5)	20/159 (12.6)	0.344

Variables are expressed as numbers and percentages or median with 25th and 75th quartiles.

AMI, acute myocardial infarction, CABG, coronary artery bypass grafting, CKD, chronic kidney disease, COPD, chronic obstructive pulmonary disease, CPR, cardiopulmonary resuscitation, LM, left main, LAD, left anterior descending, LVEF, left ventricular ejection fraction, mAFP, microaxial flow pump, PAD, peripheral artery disease, PCI, percutaneous coronary intervention, tMCS, temporary mechanical circulatory support, V‐A ECMO, veno‐arterial extracorporeal membrane oxygenation.

### Primary and secondary outcomes

**Figure 1:**
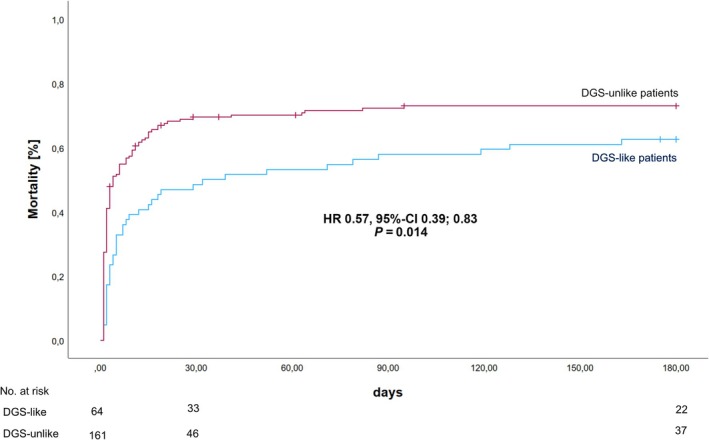
Kaplan–Meier analysis showing all‐cause 180‐day mortality comparing DanGer Shock‐like versus DanGer Shock‐unlike patients. The hazard ratio (HR) is adjusted for age, sex, atrial fibrillation, CPR before mAFP, and baseline lactate (≥2.5 mmol vs. <2.5 mmol/L).

**Figure 2:**
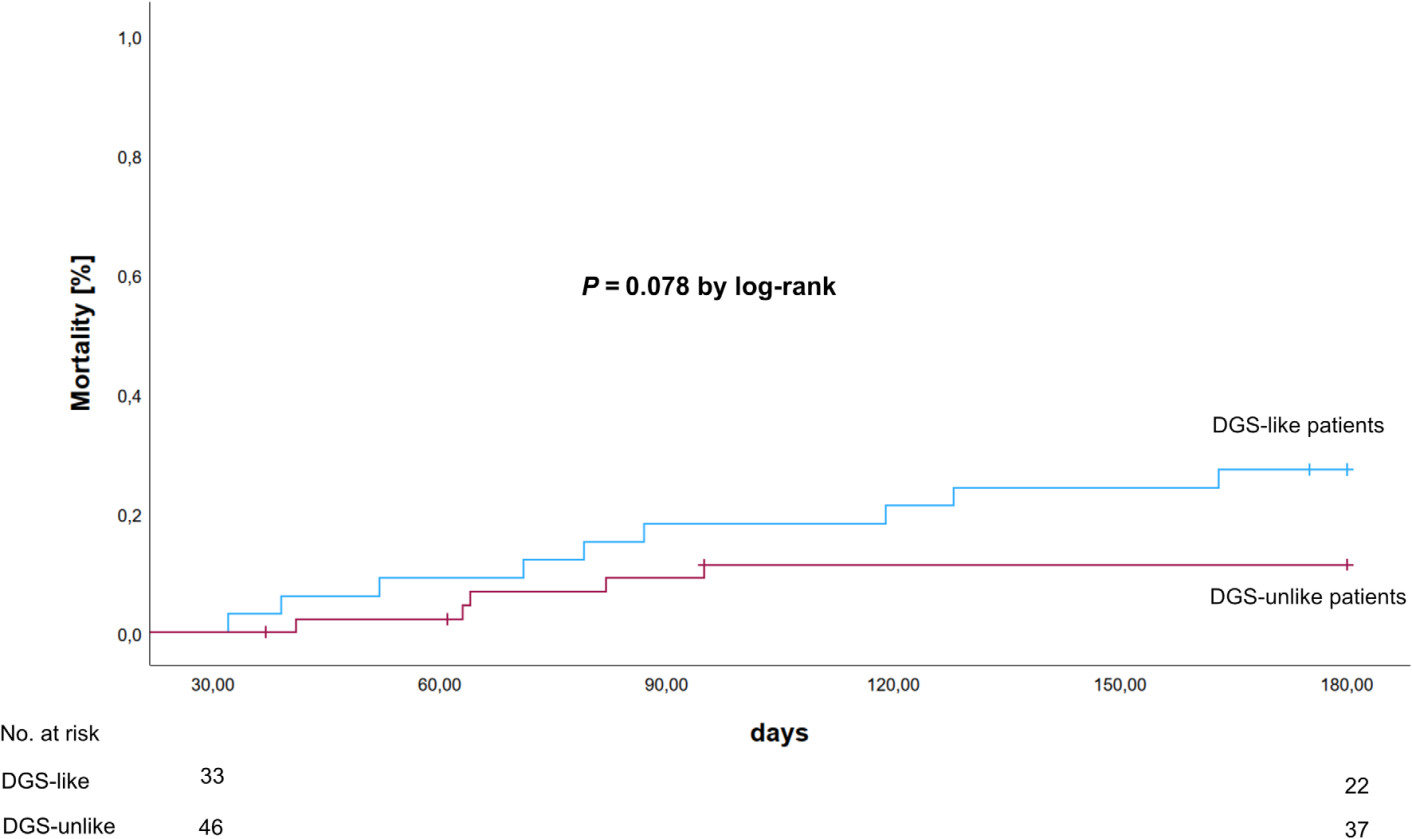
Landmark Kaplan–Meier analysis showing unadjusted all‐cause 180‐day mortality starting at day 31 comparing DanGer Shock‐like versus DanGer Shock‐unlike patients.

The primary outcome, all‐cause mortality at 180 days, was significantly lower in DGS‐like compared to DGS‐unlike (62.5% vs. 72.0%, *P* = 0.014) (*Figure* *1*). Moreover, 30‐day all‐cause mortality was lower in DGS‐like compared with DGS‐unlike (48.4% vs. 70.2%, *P* < 0.001). After adjusting for age, sex, atrial fibrillation, CPR before mAFP, and baseline lactate (≥2.5 mmol vs. <2.5 mmol/L), DGS‐like remained an independent predictor of both 180‐day (HR 0.57, 95% CI 0.39, 0.83) and 30‐day all‐cause mortality (HR 0.48, 95% CI 0.32, 0.72). Noteworthy, in a Landmark analysis starting at day 31, DGS‐like patients had a numerically higher mortality than DGS‐unlike patients (17.3% vs. 10.9%, *P* = 0.078) (*Figure* *2*).

Other secondary outcomes favoured the DGS‐like cohort with a lower rate of CPR after mAFP initiation and a lower rate of death on device, whereas weaning success was numerically higher in DGS‐like but not significantly different between groups after adjustment for the aforementioned variables (*Table* *2*). The ICU stay tended to be longer in DGS‐like patients (6 vs. 4 days).

**Table 2:** Primary and secondary outcomes.

	All *n* = 225	DGS‐like STEMI‐CS *N* = 64	DGS‐unlike STEMI‐CS *N* = 161	Adjusted coefficients (95%CI)	*P* value
Death at 180 days, *n* (%)	156/225 (69.3)	40/64 (62.5)	116/161 (72.0)	0.57 (0.39, 0.83)^a^	0.014 (log‐rank)
Death at 30 days, *n* (%)	144/225 (64.0)	31/64 (48.4)	113/161 (70.2)	0.48 (0.32, 0.72)^a^	<0.001 (log‐rank)
CPR after device, *n* (%)	119/219 (54.3)	24/63 (38.1)	95/161 (60.9)	0.50 (0.27, 0.95)^b^	0.002
Death on device, *n* (%)	100/225 (44.4)	20/64 (31.3)	80/161 (49.7)	0.36 (0.19, 0.69)^b^	0.012
Weaning success, *n* (%)	104/225 (46.2)	36/64 (56.3)	68/161 (42.2)	1.45 (0.68, 3.10)^b^	0.057
Days in ICU, days	5 (1, 15) *n* = 220	6 (3, 16) *n* = 62	4 (1, 15) *n* = 158	0.04 (−2.75, 4.74)^c^	0.057

Variables are expressed as numbers and percentages.

CPR, cardiopulmonary resuscitation; ICU, intensive care unit.

^a^ Hazard ratio (HR) with 95% confidence interval (CI) comparing DGS‐like vs. DGS‐unlike STEMI‐CS adjusted for age, sex, atrial fibrillation, CPR before mAFP, and baseline lactate (≥2.5 mmol vs. <2.5 mmol/L).

^b^ Odds ratio (OR) with 95% confidence interval (CI) comparing DGS‐like vs. DGS‐unlike STEMI‐CS adjusted for age, sex, atrial fibrillation, CPR before mAFP, and baseline lactate (≥2.5 mmol vs. <2.5 mmol/L).

^c^ Regression coefficient (beta) with 95% confidence interval (CI) comparing DGS‐like vs. DGS‐unlike STEMI‐CS adjusted for age, sex, atrial fibrillation, CPR before mAFP, and baseline lactate (≥2.5 mmol vs. <2.5 mmol/L).

### Safety outcomes

**Figure 3:**
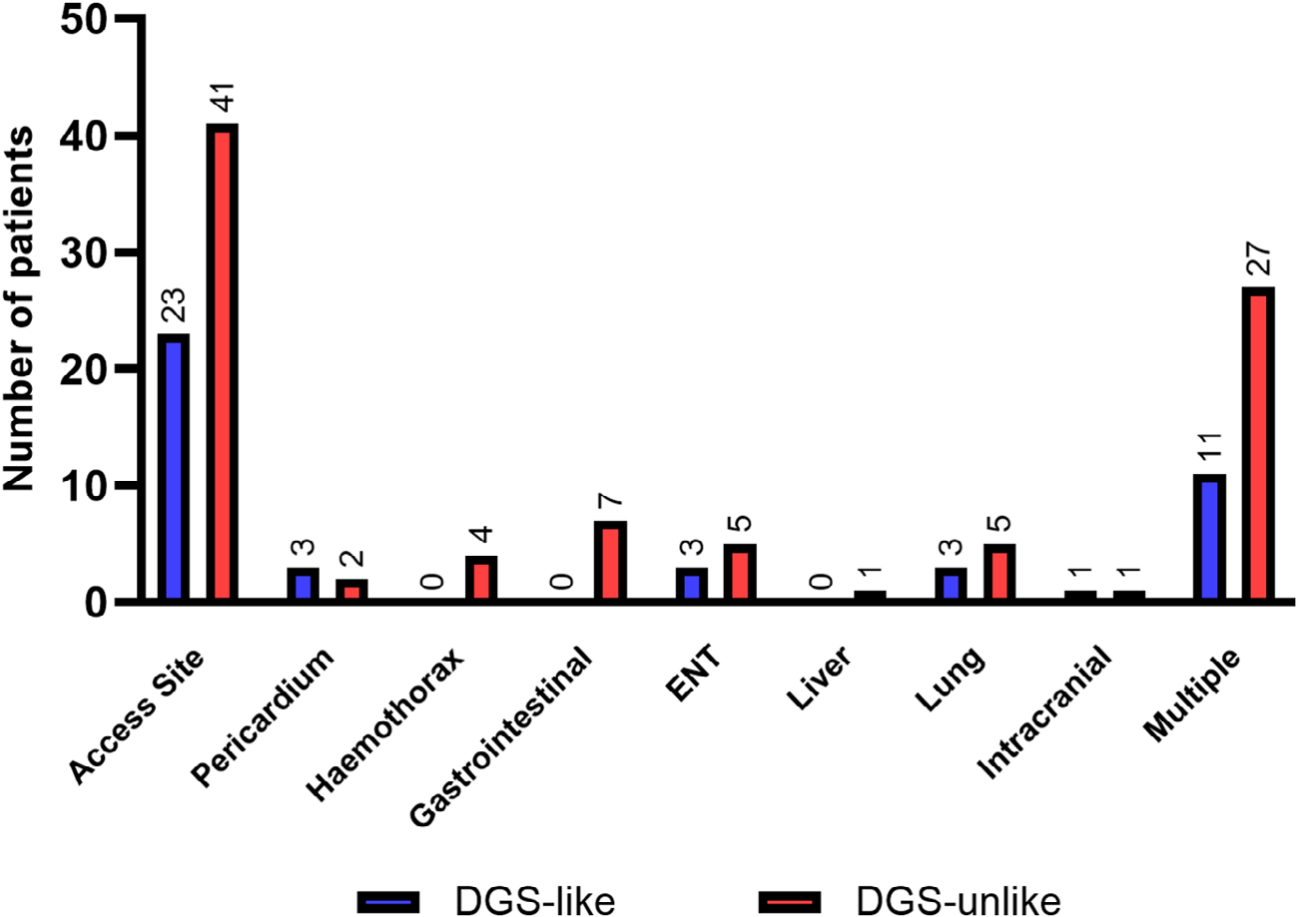
Detailed description of bleeding localization according to group in patients experiencing mild, moderate or severe GUSTO bleeding. Multiple excludes access site‐related bleeding. ENT, ear, nose, and throat.

The safety outcomes are presented in *Table* *3*. The composite of severe bleeding, limb ischaemia, haemolysis, device failure, and worsening of aortic regurgitation occurred more often in DGS‐like patients compared to DGS‐unlike patients (60.9% vs. 46.0%, *P* = 0.043). This was mainly driven by a significantly higher rate of haemolysis (43.8% vs. 26.7%, *P* = 0.013) and a numerically higher rate of limb ischaemia (10.9% vs. 3.7%, *P* = 0.054). Moreover, bleeding events were common but not different between groups. Those bleeding events were related in 53.2% to non‐access site bleeding and in 46.8% to access site bleeding (mAFP and/or PCI site) without significant differences between groups. For detailed analysis of bleeding sites, see *Figure* *3*. Noteworthy, non‐access site bleeding was associated with higher rates of GUSTO severe bleeding compared to access site bleeding (57.6% vs. 30.8%, *P* = 0.005).

**Table 3:** Safety outcomes.

	All *n* = 225	DGS‐like STEMI‐CS *N* = 64	DGS‐unlike STEMI‐CS *N* = 161	*P* value
Composite safety endpoint^a^, *n* (%)	113/225 (50.2)	39/64 (60.9)	74/161 (46.0)	0.043
GUSTO bleeding, *n* (%)				0.896
Moderate	61/225 (27.1)	16/64 (25.0)	45/161 (28.0)	
Severe	50/225 (22.2)	15/64 (23.4)	35/161 (21.7)	
Bleeding location, *n* (%)				0.294
Access site	52/111 (46.8)	17/31 (54.8)	35/80 (43.8)	
Non‐access site	59/111 (53.2)	14/31 (45.2)	45/80 (56.3)	
Limb ischaemia, *n* (%)	13/225 (5.8)	7/64 (10.9)	6/161 (3.7)	0.054
Haemolysis, *n* (%)	71/225 (31.6)	28/64 (43.8)	43/161 (26.7)	0.013
Device failure, *n* (%)	0	0	0	n.a.
New or worsening AR, *n* (%)	10/225 (4.4)	5/64 (7.8)	5/161 (3.1)	0.152
Stroke, *n* (%)	10/225 (4.4)	4/64 (6.3)	6/161 (3.7)	0.476
Cause of stroke, *n* (%)				
Ischaemic	7/10 (70.0)	4/4 (100)	3/6 (50.0)	
Haemorrhagic	3/10 (30.0)	0	3/6 (50.0)	
Sepsis, *n* (%)	32/225 (14.2)	9/64 (14.1)	23/161 (14.3)	0.966
Blood culture positive sepsis, *n* (%)	19/225 (8.4)	7/64 (10.9)	12/161 (7.5)	0.417
CVVHDF, *n* (%)	71/224 (31.7)	21/64 (32.8)	50/160 (31.3)	0.820

Variables are expressed as numbers and percentages.

AR, aortic regurgitation; CVVHDF, continuous veno‐venous haemodiafiltration; GUSTO, Global Use of Strategies to Open Occluded Arteries.

^a^ Composite of severe bleeding, limb ischaemia, haemolysis, device failure, and worsening of aortic regurgitation.

There was no case of device failure and new/worsening aortic regurgitation was infrequent. Stroke occurred in 4.4%, blood culture‐positive sepsis occurred in 8.4%, and continuous veno‐venous haemodiafiltration was performed in 31.7%, all without significant differences between groups (*Table* *3*).

In an additional Cox regression model that included clinically important characteristics known to be associated with outcome in cardiogenic shock and the aforementioned complications, none of these complications remained an independent predictor of 180‐day all‐cause mortality. However, factors like age, mean arterial blood pressure, baseline lactate, need for invasive ventilation, and DGS‐like vs. DGS‐unlike remained associated with 180‐day all‐cause mortality in this model (*Table* *S1*).

## Discussion

The main findings of the present analysis can be summarized as follows. First, approximately one‐third of patients presenting with STEMI‐CS would have been potentially eligible for inclusion in the DGS trial. Second, these patients were younger, received CPR less often, and had longer support from the mAFP. Third, DGS‐like patients had a lower all‐cause mortality at both 30 and 180 days and being DGS‐like remained independently associated with a lower mortality at both time points in a multivariable Cox regression analysis. Fourth, complications during mAFP treatment and throughout the hospital course were common; however, mortality did not appear to be predicted by these complications but by patient characteristics and markers of disease severity.

The relatively low rate of being DGS‐like (28.4%) in a cohort of patients with STEMI‐CS is in line with other reports. Schrage *et al*. reported a rate of 24.4% of patients who would have been eligible for inclusion in DGS in a cohort of 635 patients presenting with CS caused by acute myocardial infarction.^13^ Recently, an analysis of four major randomized trials investigating the impact of ECMO in CS found that in 538 patients with sufficient data to determine DGS‐like status, 202 patients (37.5%) were DGS‐like and 336 patients (62.5%) were DGS‐unlike.^14^ Moreover, a recent analysis from the Critical Care Cardiology Trials Network, which collects data from CS patients regardless of treatment in the United States and Canada, found that 32% of the patients with STEMI‐CS would meet the eligibility criteria for DGS.^15^ Importantly, in our current analysis, we did not include patients randomized to mAFP in the DGS trial at our centre to avoid double reporting and to maintain the integrity of the trial data. Therefore, we may slightly underestimate the number of DGS‐like patients.

In our analysis, being DGS‐like resulted in a younger patient cohort with lower rates of CPR and longer mAFP support. In particular, age is known to influence the outcome in CS, and advancing age has been repeatedly associated with higher mortality in patients with CS, regardless of shock severity according to the SCAI Shock classification.^16^, ^17^, ^18^, ^19^ Furthermore, in the SHOCK trial, the benefit of early revascularisation on mortality was seen only in younger patients.^5^ Beyond that, an age‐dependent effect of mAFP treatment was observed in the DGS trial, with older STEMI‐CS patients having a high mortality and no significant benefit from routine treatment with a mAFP compared with younger patients.^20^ Moreover, the exclusion of comatose OHCA but inclusion of IHCA with a maximum of 10 min to ROSC to mimic medically witnessed CA resulted in significantly lower CPR rates in the DGS‐like cohort. It seems reasonable to define resuscitated patients with limited risk of brain injury by a maximum of 10 min of CPR, as two analyses, one in a pure ECMO cohort,^14^ and another in a mixed cohort of mAFP‐treated and ECMO‐treated patients,^9^ showed a trend towards or even a significant reduction in 6‐month mortality with the use of tMCS in these patients. Overall, these observations suggest that appropriate patient selection may play an important role and that DGS‐like appears to identify a patient cohort with a low risk of hypoxic brain injury who may benefit from the routine use of mAFP in STEMI‐CS.

In our cohort, this patient selection resulted in a significantly lower mortality in DGS‐like compared to DGS‐unlike patients at both 30 and 180 days, and DGS‐like was independently associated with a lower mortality at both time points independent of age and sex and three significantly different baseline characteristics (baseline lactate ≥2.5 mmol/L, atrial fibrillation, and CPR before mAFP). In addition, a second Cox regression model including more treatment factors and complications showed similar results, but the 95% CI was wider due to the inclusion of more factors with the same number of events. Notably, mortality in our whole cohort and mortality in the DGS‐like cohort were higher than in the DGS trial,^8^ and it was also higher than in ECLS Shock^7^ or subsequent meta‐analysis of these trials and others.^9^ In contrast, observational data predicted a 30‐day mortality of 63.5% in patients eligible for DGS, which is higher than the observed 30‐day mortality of 48.4% in our cohort.^13^ These observations highlight differences between results derived from RCTs and real‐world registries in general^21^ and specifically in cardiogenic shock.^22^ The latter analysis revealed a very similar 30‐day mortality of 45.9% compared to our data in 133 617 patients presenting with AMI‐CS included in 12 registries.^22^ In contrast, 30‐day mortality was 39.9% in 2154 patients examined in 14 RCTs.^22^ These differences are most likely explained by differences in the patient population and the treatment. In our analysis and compared with the DGS trial,^8^ patients were basically comparable with regard to age, sex, arterial lactate level, and PCI treatment; however, they had more comorbidities including hypertension, diabetes, previous myocardial infarction, and chronic kidney disease, and they were more likely to have received CPR before mAFP and had higher rates of mechanical ventilation. Both RCTs and registries are scientifically and clinically important and can be considered complementary. Registries document the treatment and outcomes of patients in everyday clinical practice.

Moreover, DGS‐like patients exhibited a specific pattern of mortality, with not only a significant mortality burden up to 30 days but also, as shown in our landmark analysis, a clinically meaningful mortality between days 31 and 180. This pattern was also observed in the DGS trial.^8^ Another analysis from the National Cardiogenic Shock Initiative also found significant mortality in AMI‐CS patients beyond day 30.^19^ We can only speculate about the reasons for this finding. First, it may be related to a survival bias with more vulnerable patients in the DGS‐like group after day 30. Second, the lower rate of resuscitated patients in the DGS‐like cohort results in a distinct phenotype of CS with hypoperfusion driven primarily by low cardiac output, in contrast to comatose resuscitated patients, whose high lactate levels reflect low flow during resuscitation rather than solely poor circulation on admission.^23^ This could result in a heart failure population known to be at increased risk of death beyond the acute phase.^24^ Third, it is not only the mortality of AMI‐CS patients who survive the initial event that is of concern but also the high rehospitalization rate among survivors, which puts patients at risk of further complications contributing to the observed mortality pattern.^25^ Further research and a longer follow‐up of the DGS patients would be desirable to better understand the long‐term outcomes of these patients and the mechanisms behind, such as recovery by left ventricle unloading as currently being investigated in the STEMI‐DTU RCT in patients presenting with large anterior ST‐elevation myocardial infarction in SCAI class A/B.^26^

Concomitant administration of vasopressors and inotropes was common in our cohort with numerically lower rates in DGS‐like compared to DGS‐unlike patients; however, all *P* values were >0.10. Therefore, we cannot prove a real difference and the observation may be due to chance. In DGS, the use of a mAFP reduced the use of vasopressors and inotropes while maintaining haemodynamic stability and achieving faster normalization of lactate levels in patients with STEMI‐CS.^27^

Despite high complication rates in the overall cohort and in some parameters even higher rates in DGS‐like patients, mortality was lower in DGS‐like patients compared with DGS‐unlike patients. This mortality benefit despite an increased risk of complications was observed in the DGS trial,^8^ an individual patient data meta‐analysis of mAFP‐treated and ECMO‐treated patients,^9^ and a trend towards improved survival was observed in an analysis of DGS‐like patients in an ECMO cohort^14^ We observed higher rates of haemolysis and limb ischaemia in DGS‐like compared with DGS‐unlike patients, which contributed to the increased rate of the composite safety endpoint. This might be explained by a longer mAFP support in this cohort, which puts patients at increased risk of developing haemolysis and limb ischaemia, combined with a survival benefit in DGS‐like patients. However, the endpoint of haemolysis in particular must be interpreted with caution as it was primarily based on lactate dehydrogenase measurement with two values above 16.67 μM within 24 h and haptoglobin was only available in a minority of patients. Bleeding complications were frequent in both groups, and approximately 50% of these bleeding events were non‐access site related, highlighting the high‐risk patient population requiring intensive care treatment and bleeding related to gastrointestinal origin, haemothorax, and diffuse blood loss during the course of treatment. Notably, non‐access site‐related bleeding was more severe than access site‐related.

The rate of renal replacement therapy was lower in our cohort of patients compared to the treatment arm of the DGS trial^8^ and did not differ between DGS‐like and DGS‐unlike. In DGS, the use of mAFP was shown to be an independent factor, in addition to bleeding and shock severity, for the occurrence of acute kidney injury and the need for renal replacement therapy.^28^ It is therefore not surprising that in our cohort, where all patients received mAFP support, no difference between the groups was evident. Unfortunately, the primary reason for renal replacement therapy, for example, metabolic, acute kidney injury, or volume overload, was not recorded in our registry, and, therefore, we can only speculate about the differences in the use of renal replacement therapy in our cohort compared to the DGS trial. It is likely that factors related to the patient profile, but also treatment preferences according to patient authorisation, and individual decisions by the treating physicians may have contributed to this finding.

Hypothetically, a further reduction in these complications could be associated with improved outcomes. Device iterations with improved haemocompatibility and smaller sheath size, as well as education of the shock team on best practices, will be important to reduce complications. Notably, the complications studied in our analysis were not associated with 180‐day mortality. This must be interpreted with caution, as all patients received a mAFP and were at risk of developing complications, which is different from an analysis with a group receiving a mAFP compared to another group without a mAFP.

### Limitations

Our analysis has limitations. First, this is an observational, consecutive, single‐centre registry with all the biases inherent in a retrospectively evaluated, unmonitored registry. In particular, the derivation of these data from a single centre limits the applicability to other centres and healthcare systems, as institutional pathways and treatment algorithms may influence the results. Second, our analysis may overestimate the eligible population because some parameters were not recorded in the database, for example, right ventricular failure at baseline or non‐cardiac comorbid conditions with a life expectancy of less than 1 year, which were exclusion criteria in DGS.^8^ Third, despite applying standardized definitions, in particular for bleeding, those events were retrospectively adjudicated with all the limitations associated. Fourth, the number of patients was small, which limits the power of statistical analysis; however, due to the high event rate, multivariable models appear to be robust. Fifth, the inclusion period was long but comparable to that of the DGS trial.^8^ Sixth, only CS patients treated with a mAFP were included in this registry, which does not cover all CS patients treated at our centre. However, our primary aim was not to investigate eligibility among all CS patients, but to study the effect of patient selection in a cohort of CS patients treated with mAFP, which was indicated at the discretion of the operator. Against this background, it was also not our aim to make a statement about the treatment effect of mAFP in STEMI‐CS *per se*, as this is not possible in a cohort of patients who have all received mAFP.

## Conclusions

A DGS‐like patient profile was associated with a lower all‐cause mortality at 180 days compared with a DGS‐unlike profile in a STEMI‐CS cohort treated with mAFP, suggesting that these criteria may help to identify patients with a better prognosis in mAFP‐supported STEMI‐CS and, conversely, to recognize futile patients.

## Conflict of interest

NM reports a research and an educational grant from Abiomed to his institution, outside the submitted work; received an educational grant from Boston Scientific to his institution, outside the submitted work; and received personal fees from Edwards Lifesciences, Medtronic, Biotronik, Novartis, Sanofi Genzyme, AstraZeneca, Pfizer, Daiichi Sankyo, Abbott, Abiomed, B. Braun, and Boston Scientific, outside the submitted work. EBW reports a research grant from Boehringer‐Ingelheim and personal fees from Amgen, AstraZeneca, Bayer, Bristol Myers Squibb, Boehringer‐Ingelheim, CVRx, Daiichi Sankyo, Pfizer, and Novartis outside the submitted work. FJW reports personal fees from Abbott, Abiomed, Boston Scientific, Neovasc, Asahi, Biotronik, and Corvia Medical, outside the submitted work. SH reports personal fees from Edwards Lifesciences, Boston Scientific, Cardiac Dimensions, and Bayer, outside the submitted work. TN reports a lecturer fee from Abiomed, outside the submitted work. AL reports grant support from Edwards Lifesciences, speaker honoraria from Abbott, Abiomed, AstraZeneca, Bayer, Boston Scientific, Corvia, Daiichi Sankyo, Edwards Lifesciences, Medtronic, Meril, Novartis, Pfizer, and MSD, and owns stock options from Picardia, Transverse Medical, and Filterlex, outside the submitted work. The other authors report no conflict of interests.

## Funding

None.

## Supporting information


**Table S1.** Predictors of 180‐day all‐cause mortality derived from baseline characteristics and safety events.
