# Supplementary material for: DanGer Shock‐like profile predicts the outcome in ST‐elevation myocardial infarction‐related cardiogenic shock
Source: ESC Heart Fail. 2025 Mar 19;12(4):2759–68. doi: 10.1002/ehf2.15269 (PMC12287854; doi:10.1002/ehf2.15269)
Supplement: Supplementary file 1 — Table S1. Predictors of 180‐day all‐cause mortality derived from baseline characteristics and safety events. [file EHF2-12-2759-s001.docx]

**Supplement**

**Table S1.** Predictors of 180-day all-cause mortality derived from baseline characteristics and safety events.

|  | **Univariable** | | **Multivariable** | |
| --- | --- | --- | --- | --- |
|  | **HR (95%-CI)** | **p-value** | **HR (95%-CI)** | **P-value** |
| Age (per 1-year increase) | 1.02 (1.01; 1.04) | 0.002 | 1.02 (1.01; 1.03) | 0.008 |
| Female sex, n (%) | 1.30 (0.91; 1.86) | 0.143 | 1.24 (0.84; 1.83) | 0.272 |
| Mean blood pressure (per 1 mmHg increase) | 0.99 (0.98; 0.99) | <0.001 | 0.99 (0.98; 1.00) | 0.017 |
| Heart rate (per 1 bpm increase) | 1.00 (1.00; 1.01) | 0.845 |  |  |
| Arterial lactate (<2.5 vs. ≥2.5 mmol/l) | 1.07 (1.04; 1.10) | <0.001 | 2.16 (1.35; 3.46) | 0.001 |
| LVEF (per 1% increase) | 1.00 (0.98; 1.01) | 0.699 |  |  |
| Resuscitation before mAFP | 1.51 (1.10; 2.08) | 0.011 | 0.92 (0.62; 1.36) | 0.661 |
| 2/3-vessel disease | 1.18 (0.79; 1.75) | 0.427 |  |  |
| Infarct-related artery LM or LAD | 1.31 (0.95; 1.82) | 0.105 |  |  |
| Time from symptoms to mAFP (per 1-hour increase) | 1.00 (1.00; 1.01) | 0.700 |  |  |
| Additional tMCS | 1.78 (1.01; 3.16) | 0.047 | 1.57 (0.83; 2.99) | 0.169 |
| Invasive ventilation | 2.61 (1.53; 4.46) | <0.001 | 2.15 (1.25; 3.72) | 0.006 |
| DGS-like vs. – unlike | 1.53 (1.07; 2.20) | 0.020 | 0.68 (0.47; 1.00) | 0.05 |
| GUSTO moderate/severe bleeding | 0.82 (0.60; 1.12) | 0.211 |  |  |
| Limb ischemia | 1.07 (0.57; 2.04) | 0.827 |  |  |
| Haemolysis | 0.62 (0.44; 0.88) | 0.007 | 0.71 (0.49; 1.03) | 0.069 |
| New/worsening AR | 0.29 (0.09; 0.89) | 0.031 | 0.92 (0.28; 3.05) | 0.885 |
| Stroke | 0.45 (0.17; 1.21) | 0.448 |  |  |
| Sepsis | 0.72 (0.45; 1.16) | 0.178 |  |  |
| CVVHF | 1.17 (0.84; 1.62) | 0.358 |  |  |

Variables are expressed as hazard ratios (HR) and 95% Confidence Intervals (CI). LVEF indicates left ventricular ejection fraction; mAFP, microaxial flow pump; tMCS, temporary mechanical circulatory support; DGS, DanGer Shock; GUSTO, Global Use of Strategies to Open Occluded Arteries; AR, aortic regurgitation; CVVHF, continuous veno-venous haemofiltration.
